# Factors associated with the attraction and retention of family and community medicine and nursing residents in rural settings: a qualitative study

**DOI:** 10.1186/s12909-023-04650-1

**Published:** 2023-09-13

**Authors:** G. Tort-Nasarre, Josep Vidal-Alaball, M. J. Fígols Pedrosa, L. Vazquez Abanades, A. Forcada Arcarons, J. Deniel Rosanas

**Affiliations:** 1 Department of Nursing, Faculty of Nursing and Physiotherapy, University of Lleida, C/Montserrat Roig, Lleida, 25198 Spain; 2 SAP ANOIA. Gerencia Territorial Catalunya Central, Institut Català de La Salut, Igualada, 08700 Spain; 3 Unitat de Suport a la Recerca de la Catalunya Central, Fundació Institut Universitari per a la Recerca a l’Atenció Primària de Salut Jordi Gol i Gurina (IDIAPJGol), Sant Fruitós del Bages, 08272 Spain; 4 Health Promotion in Rural Areas Research Group, Gerencia Territorial de la Catalunya Central, Institut Català de la Salut, Sant Fruitós de Bages, 08272 Spain; 5 University of Vic-Central University of Catalonia, Vic, 08500 Spain; 6 Unitat Docent Multiprofessional d’AFiC Catalunya Central, Gerència Territorial de la Catalunya Central, Institut Català de la Salut, Sant Fruitós de Bages, 08272 Spain; 7 Gerència Territorial de la Catalunya Central, Institut Català de la Salut, 13-15, Sant Fruitós de Bages, 08272 Spain

**Keywords:** Residency programmes, Retention, Family medicine, Family nursing and community care, Primary health, Rurality.

## Abstract

**Background:**

The current shortage of primary care doctors and nurses is causing difficulties in replacement, and this shortage is expected to increase. This situation is more pronounced in rural environments than in urban ones. Family and community care specialty training is a key component of both the transition to clinical practice and the retention of new professionals. The aim of this study is to explore the attitudes and perceptions of internal medicine residents and internal nurse residents trained in a rural teaching unit on factors associated with recruitment and retention, including the role of the specialty training programme.

**Methods:**

A qualitative study was conducted. Purposive sampling was used, and thirteen residents from the central Catalonia teaching unit who were in their final year of training participated in semistructured interviews. The data were collected during 2022 and were subsequently analysed with thematic analysis. The study is reported using the COREQ checklist.

**Results:**

Six themes emerged from data related to perceptions and attitudes about the factors associated with recruitment and retention: *training programme, characteristics of the family and community specialty, concept of rural life, family and relational factors, economic and resource factors*, and *recruitment and job opportunities.*

**Conclusions:**

Family and community medicine and nursing residents trained in rural settings expressed satisfaction with the specialty programme and most features of primary care, but they experienced a wide range of uncertainties in deciding on their professional future in terms of living in rural areas, family support, financial support and recruitment. This study identifies individual and structural factors that could be of great use to retain doctors and nurses in rural areas.

## Introduction

The shortage of health professionals in rural areas is a growing problem in many countries. This situation has important implications for the quality of health care provided in these areas as well as for the health of the people who live there. Lack of access to medical care, deficiencies of health personnel and a lack of organisation and coordination of services are barriers to access and the quality of health care in these areas.

The insufficient number of trained professionals and the growing shortage of doctors and nurses causes difficulties in replacement in primary care, and this shortage is forecasted to increase [1, 2]. The WHO proposed as a global strategy the need to prioritise policies to improve the recruitment, development and retention of health care workers [3, 4].

In recent years, the primary care specialty has been increasingly unattractive for some doctors’ specialisation [5]. There are important elements to consider when primary care residents choose not to join the primary care workforce after completing residency [6] or to retain physicians who have been working in primary care [7]. In nursing, there is a shortage of generalist nurses worldwide; however, there is an increase in the retention rates of new graduates who participate in residency programmes [8].

There is a wide range of initiatives and recommendations to promote the recruitment and retention of doctors and nurses in primary and community care. The WHO recently published a systematic review on health workforce retention in remote areas [9] and guidelines on health workforce development, attraction, recruitment and retention in rural and remote areas [10]. Among the recommendations, it is worth mentioning the establishment of specific education and training programmes for health personnel working in rural and remote areas to meet the needs of the rural population.

Strategies to retain doctors and nurses include ensuring professional growth, providing minimum and uniform economic incentives across areas, and ensuring good availability of social services and economic opportunities [11]. Primary care doctor retention has a strong relationship with knowledge acquisition, empathic personality profile and the possibility of continuous personal development in addition to factors related to adequate infrastructure, organisational climate and salary [7]. In Spain, security and stability, development opportunities within the organisation, learning opportunities and the degree of autonomy are seen as more important than aspects such as remuneration [12]. Concern for the retention of new nursing professionals has been studied through evaluation of the effectiveness of nurse residency programmes (NRPs), and specialty recognition, gratification, and environment-dependent relatedness have been identified as factors for retention [13, 14].

In rural areas, attraction factors go beyond financial incentives; future professionals also value the quality of life in the rural environment, community support, nonmonetary incentives, previous family or professional experiences in a rural environment, autonomy and good professional-patient relationships [15]. Rural generalist medicine programmes are a strong point in the joint efforts to coordinate and strengthen the response to both the shortage of professionals and health needs in rural and remote areas [16, 17]. In nursing, there are also broad and multifaceted factors that influence the recruitment and retention of practising nurses in rural areas [18]. Although there are several proposals from educators, practice administrators and political leaders to address this problem, the literature in this area is still developing, and there is a lack of robust studies that specifically focus on the retention of practising nurses [19]. Kaplan et al. argued that it is too early to draw definitive conclusions regarding the ability of rural nurse practitioner residencies to foster nurse practitioners’ involvement and investment in rural communities. Further research is necessary to assess their potential long-term impact on rural primary care practice [20]. To summarise, addressing the challenges in rural areas calls for fresh ideas and strategies that differ from those applied in urban areas and that consider primary health care across the entire system. Furthermore, it is crucial to translate empirical research findings into effective actions that can address the current issues.

Studies have reported that an organised, well-funded, rural placement or rural clinical school programme produces positive associations with increased rural intentions and actual rural employment for graduates [21] and that rural clinical rotations at universities influence rural medical career choices [22, 23] but require further refining [24].

Effective training in various health science specialties, such as family and community care, plays a vital role in preparing professionals for clinical practice and retaining them in their fields. Family and community medicine and nursing specialty training programmes are common in the training systems of many countries but diverge in specific aspects. In Spain, the training for family and community medicine and nursing includes clinical skills and training in ethical values, commitment to patients, professional commitment to the specialty and to the National Health System, empathy and communication skills, new technologies and other aspects that may contribute to quality professional practice [25, 26]. Both programmes include a training stay in a rural primary care setting.

Multiprofessional Teaching Units of Family and Community Care are responsible for the quality training of future doctors and nurses as well as for the promotion of the teaching and research profile. The Multiprofessional Teaching Unit of Family and Community Care of Central Catalonia has a diverse typology of centres in accordance with the characteristics of the region itself. Some of the centres are located in an urban environment, while the vast majority of them are located in rural areas with a population of less than 7500 inhabitants, a density of 100 inhabitants/km2 and specific population characteristics [27].

In Spain, the attraction and retention of health professionals in rural areas is also a major challenge. According to data from the General Council of Official Colleges of Physicians of Spain, in 2020, only 6.7% of registered doctors in Spain worked in rural areas, even though these areas represent approximately 30% of the Spanish territory and have significant populations requiring medical care (15.9% of the Spanish population was registered in rural municipalities in 2020) [28, 29]. In Catalonia (Northwest Spain), the recruitment and retention situation of health professionals in rural areas is similar to that of the rest of Spain. In 2020, only 5.3% of registered doctors worked in municipalities with fewer than 5,000 inhabitants [30]. There are no studies in the territory that have analysed the reasons why, despite high satisfaction in the specialty programme [31], there is low retention. Knowing the reasons, motives and experiences of resident doctors and nurses who train in family and community care is useful when making recommendations for the implementation of effective strategies to improve the retention and loyalty of these professionals in the region where they completed their residency.

This study can provide valuable information on the factors that influence the decision of health care residents to work in rural areas as well as on the strategies that can be used to retain them. In addition, it can provide a complementary and novel perspective since the perspectives of doctors and nurses are combined in a single investigation, which can enrich the understanding of the phenomenon.

This study aims to explore the attitudes and perceptions of internal medicine residents and internal nurse residents trained in a rural teaching unit on factors associated with recruitment and retention, including the role of the specialty training programme.

## Methodology

### Design

A qualitative study was conducted to explore and understand the issues based on the individual experiences of the participants. This design is suitable for obtaining a deeper understanding of practice in applied disciplines and is especially relevant when the goal is to understand the perspective and experience of participants [32]. It also allows us to obtain research data in a specific context [33].

### Participants

The participants were resident doctors and nurses who were in their final year of training in family and community medicine from the teaching unit of central Catalonia. There were no exclusion criteria. Participants were selected using purposeful sampling [34] based on pragmatic and convenience criteria such as feasibility, interest and time until data saturation was obtained [35]. Participants of different ages, genders and geographic backgrounds were included to cover all ranges of experience [36].

### Data collection

Data were collected using semistructured interviews. The research team prepared a set of interview questions relevant to the objectives of the study, including the following questions: Based on your experience, what barriers and facilitators do you identify regarding recruitment and retention in the centres of central Catalonia? In your opinion, how has the training process been in the family and community residency?

Recruitment of participants was carried out by the teaching unit that had data on the final-year residents. From these records, students were approached according to the criteria of access, their interest in participating and the relevance of the study topic to their experience. All residents (n = 29) were invited to participate, and 13 residents agreed to be included in the study. Residents who showed interest in participating were contacted by telephone to schedule the interview. Initially, 11 participants were interviewed. Then, two additional participants were interviewed until data saturation was achieved (n = 13), as we needed to ensure that we gathered sufficient information and insights for a comprehensive understanding of the research topic.

The interviews were carried out by the PI between April and May 2022. We chose to conduct interviews via videoconference due to the wide dispersion of the region and to facilitate accessibility. The interviews were recorded with the permission of the participants using the Microsoft Teams communication platform provided by the Catalan Institute of Health. During the interviews, follow-up questions were asked to encourage participants to provide additional details about their perspective. Participants were recommended to participate in the interview in a quiet place without interruptions to ensure confidentiality. The interviews lasted a minimum of 35 min and a maximum of 55 min. The interviews were conducted in Catalan or Spanish, the two official languages of Catalonia, and transcribed into Catalan. Subsequently, the interviews were returned to the participants for approval of the content. All of the participants accepted the content of the interview.

### Data analysis

The data were analysed using thematic analysis [37] supported by Atlas.ti v. 9. Patterns were identified in the collected data, and themes were organised systematically to meet the research objectives [33]. The PI was responsible for analysing the data because of her experience in qualitative analysis. Likewise, the PI maintained a constant review process with a colleague external to the team and thus ensured the validity of the study. The entire process was explained to the research team, and consensus was obtained when necessary.

The process involved several steps. First, the PI familiarised herself with the data by listening to the recordings, transcribing them, and carefully reading and rereading the transcripts. Then, the PI identified meaning units that were relevant to the research objectives, generated codes, and explored relationships among them. Next, she grouped the codes into abstract themes and defined the boundaries of each theme. The PI identified six themes, which were composed of meaning units and formed the primary structure for the analysis. Finally, the research team organised the themes and subthemes and wrote the research report.

### Rigor criteria and ethical considerations

This study met the criteria for credibility, transferability, dependability and confirmability to ensure trustworthiness in qualitative research [38]. The interviews were conducted by the PI, who was an RN, PHD in nursing. She worked in primary care and served as a lecturer in the Nursing Degree Program at the university. There was no prior relationship between the PI and the project participants. The PI had extensive experience in qualitative methodology. The PI recorded her impressions during the process of conducting the interviews and analysis to ensure maximum objectivity in the procedure. The research team made constant revisions to the analysis process to ensure qualitative validity. The COREQ checklist was used to run and evaluate the study [39].

The study was approved by the University Institute for Primary Care Research (IDIAP) Jordi Gol i Gurina Clinical Research Ethics Committee (Code 22/048-P). All participants signed an informed consent document. Data confidentiality and anonymity were ensured throughout the process by assigning each participant an alpha-numeric code.

## Results

Thirteen residents in their final year of training in the specialty of family and community medicine and nursing at the Central Catalonia Teaching Unit participated in the study. Table 1 shows the main sociodemographic characteristics of the participants. Of those interviewed, 5 (38%) stayed to work in central Catalonia.

**Table 1:** Sociodemographic data

Participant	Profession	Gender	Age		Preference in selection of specialty
P1	nurse	female	29		second
P2	nurse	female	26		first
P3	nurse	female	25		second
P4	nurse	female	25		first
P5	doctor	female	29		second
P6	doctor	female	29		second
P7	doctor	male	31		second
P8	doctor	female	29		first
P9	doctor	female	34		first
P10	doctor	female	28		first
P11	doctor	female	45		second
P12	doctor	female	28		first
P13	doctor	female	28		first

The results of this study were based on six themes that emerged in the thematic analysis of the data (Table 2).

**Table 2:** Themes and subthemes

Theme	subtheme
Factors related to the training programme	Individualised training Mentoring process Shifts and on-call duty
Factors related to the characteristics of the family and community specialty	Previous training Disrepute of family doctors Generalist specialty, independence and longitudinality Doctor‒nurse/patient relationship Patient-centred model Teamwork Bureaucracy Short patient time
Factors related to the concept of rural life	Adaptation into the community Rural lifestyle
Family and relational factors	Family ties Having a partner
Economic and resource factors	Economic incentives Cost of living
Factors related to recruitment and job opportunities.	Types of contracts Nursing specialty and loss of talent and demotivation specific pool of specialists

### Factors related to the training programme

Participants highlighted the advantages of training in a rural area, such as **individualisation** in learning.

*I chose Central Catalonia as an option. I had been told that the training of doctors was good because it has a smaller hospital and the family resident had more prominence in the different specialties, and that made me decide to come. P5*.

They had opportunities to be in a teaching unit with few residents.

*Because it is small and rural, I am the only resident, and the teaching is at an individual level, and you see everything in first person. There is very good feedback that perhaps if there were more residents would not be so personalised, and you can get involved and participate a lot in the activities of each service where you go. P5*.

*You get individual treatment. I’m not just nurse number 130, but I’m XX, and I am valued and empowered. P2*.

The majority of residents, both doctors and nurses, positively assessed **the mentoring process** for their learning.

*I have been fortunate to have a very good tutor on a personal and professional level. I have learned a lot, and she has given me a lot of confidence to discuss any questions I have. P5*.

However, they also explained how this relationship influenced retention.

*If you don’t have a good relationship with the tutor, if you have a fairly negative experience, that makes you not want to stay. P6*.

Among the learning activities in the context of professional practice, the **shifts** in different hospital clinical units were highly appreciated by both doctors and nurses.

*You learn a lot on shifts. It completes your training. And then on a day-to-day basis, because you’ve seen so much, you can deal with it. P1*.

They valued the purpose of the shifts to better understand the area’s resources.

*We have to go through the different services because we have to know what is acute or what is chronic and those that can be made acute, what we can do from the primary care centre. In the end, we are the ones who refer the different services, or we can also solve problems in the primary care centre. P5*.

They also pointed out that **doing hospital on-call duty** provided them with security to treat critical illnesses in rural areas.

*Because then if I find a serious case in the primary care centre, we have a little more back-up. Because few come to me, but when they do, it’s good to know what to do. P6*.

### Factors related to the characteristics of the family and community specialty and personal motivations

The participants explained their reasons for choosing the family specialty. All the nurses had studied primary care subjects during their nursing degree curriculum, and they explained how this **previous training experience** was crucial when choosing their specialty.

*I was going to study mental health; it was my goal but for my last internship in my fourth year I went to a rural primary care centre and there I fell in love with primary. P2*.

On the other hand, not all the doctors had taken specific primary care subjects during their university training and believed that this could later influence them in not choosing this specialty.

*You have cardiology, nephrology, and digestive medicine, but there is no family medicine. If everything that it covers were really explained, there would not be this kind of rejection, in inverted commas. I have to say that family medicine is the last thing to be chosen because it is for those who had bad results in the internal medicine residency. P8*.

The participants said that for a subject within the curriculum, it would help to have more doctors who would want that specialty.

*If a course of study were made, we would lose the idea that the family doctor is the doctor who does not have a specialty because he or she touches upon everything and knows nothing in depth. P8*.

Another characteristic that the participants considered was the **disrepute** of family doctors and how this aspect may influence the poor recruitment of family professionals.

*I think what has gone wrong is the belief that good doctors work in the hospital and bad doctors stay in primary care. P8*.

However, despite the discrediting of the specialty among the medical community and socially, this did not deter them from choosing the specialty.

*There really is a lack of prestige, and I think it comes from the previous era, when people finished their degree and were already primary care doctors without a specialty. And people have kept this particular idea. It is not global, not everyone thinks so, but there is a kind of thinking that the primary care doctor is the idiot who did not want to do a specialty. P10*.

Therefore, although many of them already had previous experience in primary care and motivations for choosing the specialty, during the residency period, they identified characteristics of primary care that could be related to retention and willingness to practice, such as the generalist **approach**.

*I wanted a specialty that covered a lot, not focusing on being an eye doctor, but something of the whole body, not just centred around one area. The variability that family medicine gives you, that you don’t know what you are going to get the next day. I like variety. P10*.

They discovered the extent of primary care as a specialty, including aspects such as professional **independence** and **longitudinality**.

*In family medicine, you are lucky enough to have the chance to specialise in one thing or another, and then you also have all this part of minor surgery techniques, injections and ultrasound scans, which is also something this allows you to do. P8.*

*We also have our independence to do things. So that’s what I take away most - I have been surprised in that sense. P3*.

*I must emphasise the importance of the relationship you have with the patient and the importance of following up with the patient, of knowing what has happened to them. I find this very important for health care because if you give them medication but the next day something happens because of the medication, for example, you give them an antihypertensive because they have low blood pressure, cramps in their legs or they sweat at night…. you can be reached, and you are accessible for them to call and say, look, I have not been doing well, or I want to stop taking the pills. Well, that is very important for you to know. P5*.

They specified the importance of the **doctor‒patient relationship** in primary care and of the holistic perspective of the person and the environment as an aspect that favours the relationship.

*I have seen the doctor‒patient relationship at a later stage. The way you relate to the patient and know their family is very important. At a social level, this has been more of a discovery now in the residency. P13*.

*Seeing the environment where the patient lives helps you to understand more about what they sometimes explain. When you see them in the environment where they live, you can understand the difficulty, the other person’s problem, the experience of living with a disease, etc. I believe that what makes family medicine special is knowing the patient’s environment. P8*.

Both doctors and nurses emphasised the **patient-centred model** in the family and community setting.

*I have seen that individualisation is kind of the essence because you know that not everything is as mechanical or as easy as the clinical practice guideline says, and it is more about adapting to the person and the context. P13*.

Furthermore, during the residency, both nurses and doctors talked about the need to improve **teamwork**.

*I think teamwork is very important for me. We are a very individualistic group, especially doctors, and we do not know much about teamwork. I have seen now that the work is very hierarchical. In other words, there has been teamwork, but more than teamwork, it has been more group work, something that is essential, that we need to incorporate in our day-to-day life and even more so in primary school. It is about sharing knowledge and different visions. We should be more decisive as a team, and I think there is still a lot to do. P13*.

They also found negative aspects in daily tasks, such as the **bureaucratic** part of being a family doctor.

*The bureaucratic part consumes a lot of your time and takes it away from the care part, and then you are also the gateway to all the frustrations of the patient with all the other specialists: if I have not been called by the traumatologist, I’ll take it out on you later. The ophthalmologist should have asked me for it, and he didn’t so I’ll take it out on you. And then there’s the issue of work discharges. I can’t deal with it. It’s beyond me. P12*.

They also felt limited by the **little time** that could be devoted to each patient.

*In practice, I have found the time per patient to be totally insufficient. I find it practically impossible to monitor chronicity in 10 min. It gives me the feeling that I’m kind of postponing things I don’t know for later, for when I have time to look at the ones I’m not resolving because I don’t have time to think anymore. For me, it creates discomfort in my day-to-day life to know that I am not doing things right and that I no longer have time to deal with things calmly. P12*.

### Factors related to the concept of rural life

The participants talked about their experience of living in a village in terms of adaptation and integration into the community.

*Here in Osona, I have integrated very well and I feel very comfortable, both with the professional team and on a personal level, and that’s why I would like to stay. P5*.

*Most come, spend the four years and eventually integrate with the other residents but not with the community, not too much.*

Some participants mentioned that they had come from a large city and were attracted to the rural environment.

*I really like the rural lifestyle. I came from a super, super big city. There, you don’t know anyone, but here in the village, I really enjoy it because you get to know people and you become part of the community. P4*.

Additionally, the participants had different opinions about the attraction of rural medicine and consequently about practising professionally in these areas.

*I like it. I prefer rural areas over the city. I suppose that because of the proximity of the patients and because it is a small team, there tends to be more communication. P6*.

*I prefer the idea of an urban area. Maybe the kind of people you have to deal with isn’t ideal, but I don’t see myself working alone in a rural area. I like the idea of having more co-workers, having a fairly large centre where there are more people and being able to talk about things. P9*.

### Family and relational factors

One aspect that stood out notably was family ties as a reason for doctors and nurses to not stay and to return home.

*They leave because they have family there. I understand it. P8*

The issue of not finding a partner in the area was also prominent for doctors and nurses.

Either you marry someone from here, or you won’t get them to stay. It is the link that would make them stay, but that is very difficult. P6.

### Economic and resource factors

Some doctors mentioned that salary was not a motivating factor in choosing the specialty.

*It is very clear to me that I did not study family medicine for the money that is earned. P3*.

However, some participants stated that **they had no economic incentives** to remain in rural areas; therefore, they preferred to go abroad where the economic conditions were better.

*Perhaps they could do it, I don’t know, as some places in the south of France do: for five years you don’t pay taxes, they offer you a house to stay in, and if you have any problem with the issue of the offices, they help you with everything. P8*.

In addition, some participants from other areas found that in the area where they studied, the cost of living was more expensive compared to the area they came from. They noted that the salary was not sufficient for living in this area.

*The rent here is super expensive. Life is expensive here compared to where I am from, and the salary is not very high either. If you equate it with what you spend on rent and everything, it’s not that much. For example, in Murcia, they do earn much more. P1*.

*I need stability and to know that I am going to have a medium- or long-term contract that will allow me to sort out my life, not to be waiting. P3*.

### Factors related to recruitment and job opportunities

The participants noted that deciding on the near future was a complex and uncertain process. This organisational situation put pressure on them to decide and was stressful.

The data showed a difference between medical and nursing staff in relation to the types of contracts. For example, doctors had more defined and clear contracts with more stable contractual conditions compared to nurses.

*In principle, the offer is good; it is interim, and from what I have felt nursing is not that lucky. Nurses have very small contracts and are constantly having to see if they will be renewed, possibly because of the need for more medical professionals who offer us better conditions. P5*.

For nurses, the job offers were lacking or included short and uncertain contracts.

*I would like to stay in the area where I am in training right now, but I am not being offered anything. I feel very sad. P4*.

*Maybe you have a chance to stay, but we can’t offer you a very big contract… they don’t give you much hope either, and you have to organise your life. I have to pay the rent and not wait to see if something falls into my lap. P3*.

For some, a medium- or long-term contract would enhance retention.

*I need stability and to know that I am going to have a medium- or long-term contract that will allow me to sort out my life, not to be waiting. P3*.

The nurses perceived a dichotomy between initial expectations and job offers after completion of the residency. They experienced this as a **loss of talent and demotivation**.

*They are doing it wrong because we are all leaving. They should act in your favour because they have already taught you and they have trained you. That’s worth money and, in the end, no, they don’t take you into account as they should. P1*.

Another issue was a loss of economic resources and a lack of expert appraisal.

*I don’t understand it because you have already been trained. You have more knowledge. And they don’t value it; they give you a contract just like that person without a specialty. It’s a waste of talent and money. P1*.

Additionally, the time lag between the job offers and having to make a decision limited retention and decreased the offer.

*Maybe people will leave anyway, but I think that a percentage would stay if they had that time, let’s say, to make a decision, because it’s not like buying a t-shirt in one colour or another. I mean, it’s choosing your future career and where you are going to live. P3*.

Additionally, some doctors stated that not being able to stay in the unit where they had been trained reduced their chances of retention.

*Our management does not offer for us to stay in our health centre but in any centre in the region, and this is a limiting factor. I think that if they offer you your own centre and your familiar environment it would be much better, but to go to a health centre that you don’t know, it means you go to your own city and start there again. P10*.

A negative aspect for nurses was the lack of specialty assessment and not having a **specific pool of specialists** to fill positions.

*We do not have a specific pool, but it is true that I believe that this assessment, this plus, should be given. I think that should be considered when it comes to actually offering something at the end. P3*.

This experience led the nurses to opt for other autonomous communities where the specialty was valued within the labour pool or where there was a specific pool of specialists.

*Many of the resident nurses end up returning to their place of origin or to other autonomous communities in Spain where the specialty is valued both economically and when it comes to opting for a position or a longer contract. P3*.

*They come back also because there are communities where nursing salaries are much higher. So, if they do have a community where they also have a specific pool, they will hire you earlier and with a better salary, and you will also be close to home. P2*.

## Discussion

This study explored the factors related to the recruitment and retention of family and community medicine residents in a rural area of Catalonia (Spain). In addition, the study aimed to determine whether the experiences of the specialty training programme had a relationship with subsequent loyalty to the area where the residents were trained.

The decision of the residents to train in this rural area of Catalonia was, for most of the participants, a consequence of the scores obtained on the internal medicine and nursing residency exams. Most internal medicine resident positions in family and community medicine and internal nursing resident positions in family and community nursing are chosen by the applicants with the lowest internal medical residency exam scores [5, 37]. The positions offered in this geographic area are the last positions chosen in Spain. 53% chose the specialty of family and community medicine as their first choice compared to 46% who had other preferences. These data coincide with other studies showing that training in family medicine is not among the best positioned preferences of students [1, 40].

The results of the study shows that the decision to work in rural areas is influenced by the convergence of various factors: the training programme, characteristics of the family and community specialty, concept of rural life, family and relational factors, economic and resource factors, and recruitment and job opportunities. These aspects identified by the participants should be considered as a whole set of intertwining factors and not in isolation since they provide a comprehensive view of the complex process involved in the decisions of future professionals.

### Factors related to the training programme

There is a significant body of research on the impact of residency training on the choice to practice in rural areas [41, 42] Since this study was conducted in Spain, the results can contribute to a better understanding of whether there are other important factors that have not been previously studied. Most of the residents defined the training programme as rewarding and having significant learning opportunities. Specifically, they highlighted the facilitating factors that allowed them to learn about the resources of the area, carry out shifts on different hospital equipment, and receive teaching during shifts. This reinforces findings from other studies [31]. The role of the tutor is fundamental because of the progressive assumption of responsibilities. The study also highlights the advantages of training in the rural environment, such as the opportunity to receive individualised training and to be part of small teams, to know the patients’ environment and thus better understand their health needs and to adapt the health resources offered by the rural environment to these needs. However, rural settings can present disadvantages for health care professionals, including smaller teams that limit diverse perspectives and knowledge exchange. Limited resources in local centres lead to fewer services and restricted access to advanced technology, specialists, and continuing education programmes. This narrow exposure to clinical situations and procedures can impact professional development.

In addition, the participants identified the personal and professional relationships established during the training period as factors to be taken into account when assessing their professional future in the environment where they were trained. This can be both an advantage and a disadvantage depending on the quality and support provided by these relationships. Therefore, it is crucial to consider the nature of these relationships when evaluating the impact on residents’ professional trajectory.

### Factors related to the characteristics of the family and community specialty

The results are consistent with other research studies that show similar findings. Previous training in primary care during undergraduate studies has been shown to be a motivating factor for choosing a specialty. In this regard, this training should be introduced into the educational curriculum in faculties where it currently has limited or no presence [43, [44].

Furthermore, general medicine may suffer from a reputation of being undervalued and less prestigious compared to specialised fields [43], which may discourage medical professionals from choosing this career path. This lack of recognition and societal appreciation may result in decreased professional satisfaction and motivation among general practitioners. Efforts should be made to promote professional development and recognition within the field to attract and retain talented individuals.

During the residency period, the participants discovered aspects of primary care that they considered to be positive for the good professional development of the specialty and that could be related to the retention and professional practice of primary care. They also commented on the presence of negative aspects, such as excessive bureaucracy in consultations and the lack of time dedicated to each patient. Other studies have also recognised these factors as negative aspects of the specialty [31, 44, 45]. In rural primary care, it is even more crucial to take measures to address the challenges related to bureaucracy, resource distribution, and patient-centred care models. These centres often face additional challenges due to the lack of infrastructure and available resources compared to urban centres.

### Factors related to the concept of rural life

Residents identified the concept of the rural lifestyle as one of the determinants when deciding on their future, in accordance with other studies [46]. Cosgrave et al. (2019) analysed the social determinants of retention in rural areas and found that fulfilling life aspirations and interest in rurality favoured retention. Students who have personal interests and values related to the rural lifestyle can overcome the perceived barriers and difficulties of being a rural doctor or nurse. Coming from a rural background is one of the strongest predictors of practising in rural areas. However, the results also suggest that coming from cities does not prevent individuals from finding rural life attractive [47].

Working on barriers as an inclusive aspect of the family and community-based programme may lead to fewer doctors opting to leave. The results of the study showed that feeling like the doctor or nurse of the town supports retention in rural areas. Establishing a meaningful relationship with patients is valued very highly, but it is not enough to improve retention [13, 47, 48]. The rural lifestyle is a negative aspect when it is seen as a barrier to retention globally [18], so further research should be conducted on aspects that can make rural life attractive to young people.

### Family and relational factors

The participants mentioned the difficulty of having family support to reconcile their personal and professional life if they stayed in rural areas after completing their residencies. These results were also identified in previous studies [14, 49]. Feelings of isolation and a lack of integration are personal factors that were identified in other studies [50], and the current results point in that direction since friendships or support networks were identified as very important and fundamental for retention. It should be noted that the impact of the COVID-19 pandemic during training influenced the ability to form bonds [51–53]. The experience of being in closed villages and the difficulty of integrating during training may have been consequences of the social impact of the pandemic. On the other hand, Handoyo et al. [54] showed that people with greater resilience perform better in rural areas.

**Economic and resource** factors.

The residents stressed that receiving economic and financial incentives could help them settle in remote areas [17, 55]. On the other hand, the cost of living in the rural area studied was high in comparison with other geographical areas in the rest of Spain. This resulted in many residents deciding to return to their area of origin where the cost of living was lower. This aspect is difficult to combat unless economic and fiscal incentive policies are put in place, such as housing subsidies, free public transport, and tax reductions for settling in rural areas. Similar strategies are currently being implemented by some governments to build pharmacist loyalty in rural areas [48]. However, for more effective retention, in addition to one-off financial strategies, personal and professional strategies that promote long-term recognition must be addressed [56].

Factors related to **recruitment and job opportunities**.

In terms of factors related to recruitment and job opportunities, there are significant differences between medical and nursing residents. While medical residents claim that they are offered the possibility of accessing stable interim contracts, the same is not true for nurses, who complain that they do not have the opportunity to access stable contracts. Nurses are among the health professionals with the lowest proportion of permanent contracts, standing at 25% during the first 4 years of employment in Catalonia [57]. Spain’s precarious employment of nurses and the emigration of nurses to work in other European countries or around the world during the last decades has been well documented in previous studies [58]. The situation for doctors is very different, as there is a high shortage of professionals, which leads them to be hired very quickly [3]. The results, however, show that better planning of the supply would help people stay in the area and would give them the opportunity to stay in the same centre where they were trained. Although the nursing specialty programmes, specifically the family nursing programme, show positive results in terms of professional skills acquired, the nursing residents themselves indicate that they do not feel sufficiently recognised because they do not have a specific score valued for entry into public health service provider companies. This exacerbates their feelings of frustration and dissatisfaction [48].

### Limitations

This study has some limitations. The study design was qualitative; therefore, it was not possible to quantify the results of the training experience and its link to retention. Furthermore, the research examined perceptions and attitudes towards retention and the possible relationship with the training programme. A future study with a mixed methodology could achieve a more detailed analysis of the aspects that could be included in the training programme to promote better acceptance of the rural environment.

The study only included residents’ perspectives. A broader investigation including other people, such as tutors, policy-makers and academics, could be useful to complement the data. Likewise, a longitudinal study could complete our knowledge of the barriers and facilitators found over time regarding professional practice in rural areas.

## Conclusions

Rural-trained family and community medicine and nursing residents are satisfied with the specialty programme and with primary care but experience a wide range of uncertainties in deciding on their professional future in terms of living in villages, family support, financial and economic support, and recruitment. They also propose the implementation of more established retention strategies. Overall, this study contributes to the literature by providing a comprehensive analysis of the factors that influence the recruitment and retention of health care professionals in rural areas, as demonstrated in the context of family and community medicine in Spain. The identification of key themes, insights into perceptions and attitudes, and consideration of individual and structural factors offer valuable knowledge to inform strategies aimed at retaining doctors and nurses in rural areas. This study opens the way for the development of interventions in the field of health management and teaching to promote better retention in rural areas where health workers have been trained and introduces ways to address the shortage of health care professionals.

## Data Availability

The data that support the findings of this study are available (de-identified) from the corresponding author upon reasonable request.
