# Correction: Factors associated with the attraction and retention of family and community medicine and nursing residents in rural settings: a qualitative study

**DOI:** 10.1186/s12909-023-04814-z

**Published:** 2023-11-02

**Authors:** G. Tort-Nasarre, Josep Vidal-Alaball, M. J. Fígols Pedrosa, L. Vazquez Abanades, A. Forcada Arcarons, J. Deniel Rosanas

**Affiliations:** 1 Department of Nursing, Faculty of Nursing and Physiotherapy, University of Lleida, C/Montserrat Roig, 25198 Lleida, Spain; 2 SAP ANOIA, Gerencia Territorial Catalunya Central, Institut Català de La Salut, 08700 Igualada, Spain; 3 Unitat de Suport a La Recerca de La Catalunya Central, Fundació Institut Universitari Per a La Recerca a L’Atenció Primària de Salut Jordi Gol I Gurina (IDIAPJGol), 08272 Sant Fruitós del Bages, Spain; 4 Health Promotion in Rural Areas Research Group, Gerencia Territorial de La Catalunya Central, Institut Català de La Salut, 08272 Sant Fruitós de Bages, Spain; 5 University of Vic-Central University of Catalonia, 08500 Vic, Spain; 6 Unitat Docent Multiprofessional d’AFiC Catalunya Central, Gerència Territorial de La Catalunya Central, Institut Català de La Salut, 08272 Sant Fruitós de Bages, Spain; 7 Gerència Territorial de La Catalunya Central, Institut Català de La Salut, 13-15, 08272 Sant Fruitós de Bages, Spain

**Correction: BMC Med Educ 23, 662 (2023)**

**https://doi.org/10.1186/s12909-023-04650-1**

Following publication of the original article [1], we have been informed that author J. Deniel Rosanas has been incorrectly affiliated.

The incorrect affiliation is: Department of Nursing, Faculty of Nursing and Physiotherapy, University of Lleida, C/Montserrat Roig, Lleida 25198, Spain

The correct affiliation is: Unitat Docent Multiprofessional d’AFiC Catalunya Central, Gerència Territorial de la Catalunya Central, Institut Català de la Salut, Sant Fruitós de Bages 08272, Spain
